# Correlates of accelerometer‐assessed physical activity in pregnancy—The 2015 Pelotas (Brazil) Birth Cohort Study

**DOI:** 10.1111/sms.13083

**Published:** 2018-04-06

**Authors:** S. G. da Silva, K. R. Evenson, I. C. M. da Silva, M. A. Mendes, M. R. Domingues, M. F. da Silveira, F. C. Wehrmeister, U. Ekelund, P. C. Hallal

**Affiliations:** ^1^ Postgraduate Program in Epidemiology Federal University of Pelotas Pelotas Brazil; ^2^ Department of Epidemiology Gillings School of Global Public Health University of North Carolina ‐ Chapel Hill Chapel Hill NC USA; ^3^ Postgraduate Program in Physical Education Federal University of Pelotas Pelotas Brazil; ^4^ Department of Sport Medicine Norwegian School of Sport Sciences Oslo Norway

**Keywords:** accelerometry, antenatal care, cohort studies, maternal‐child health, physical activity, pregnancy, pregnant women

## Abstract

Objective methods to measure physical activity (PA) have become available and widely used given the high degree of precision to evaluate PA. However, few studies have used accelerometers to measure PA during pregnancy, especially in low‐ and middle‐income countries. We assessed overall PA, moderate, vigorous, and moderate‐to‐vigorous physical activity (MVPA) objectively measured among pregnant women and their correlates in a population‐based study. PA was assessed for seven consecutive days using a raw triaxial wrist‐worn accelerometer in women interviewed around 16 and 24 weeks of gestation in the 2015 Pelotas (Brazil) Birth Cohort Study. The average acceleration, which expresses overall PA, was presented in milli‐*g* (1 m*g *= 0.001 *g*), and average time (min/day) spent in MVPA (>100 m*g*) was also analyzed in 5‐ and 10‐min bouts. Analyses were performed using linear regression. In total, 2317 women were included in the analyses. Overall PA was 27.6 m*g*. Pregnant women spent on average 14 min/day in MVPA and 0.4 min in vigorous PA. Time spent in MVPA and total PA were inversely associated with years in school and income, and were lower among women receiving advice to not exercise. MVPA was also inversely associated with age, lower among women living with a partner, and higher among non‐white women. The study indicated low levels of PA among pregnant women. The identified correlates may provide a framework to better understand factors influencing PA during pregnancy and thus inform future interventions.

## 1 INTRODUCTION

During the last two decades, a growing interest in the potential beneficial effects of physical activity (PA) during pregnancy for both the mother and her offspring has emerged. Despite the positive evidence^1^ and evolution of the guidelines to promote physical activity in pregnancy,^2^ most pregnant women in high‐income settings do not reach the current recommendations^2^ of at least 20‐30 min of exercise on most days/week.^3^

Previous population‐based studies have shown that few women engaged in leisure‐time PA (LTPA) during pregnancy^4^, ^5^, ^6^ range from 4% in Brazil^5^ to 20% in Norway.^6^ However, most prevalence estimates of PA during pregnancy are based on self‐reported measures that may be prone to misclassification due to social desirability and response bias. In addition, questionnaires tend to overestimate the prevalence of PA during pregnancy.^7^

In recent years, objective methods to measure PA, such as accelerometers, have become available and widely used given the high degree of precision to quantify the intensity and duration of PA. Nonetheless, few studies have used these methods to measure PA during pregnancy^8^ especially in low‐ and middle‐income countries.^9^ Also, existing studies evaluated specific populations with uniaxial accelerometers,^10^, ^11^ which undercount some forms of physical activity, and presented a small sample size.^8^

From 2003 to 2006, the United States (US) NHANES (*National Health and Nutrition Examination Survey*) introduced accelerometry to assess PA in the population. As the sample included pregnant women, this data source provided the first objective estimates of PA at population level in pregnant women using a uniaxial accelerometer worn at the hip.^8^ Results indicated that women engaged in on average 12 min/day of moderate activity and 0.3 min/day of vigorous activity. The average time spent participating in moderate‐to‐vigorous PA varied by trimester: 12 min/day in the first trimester, 14 min/day in the second trimester, and 8 minutes/day in the third trimester. Cut points applied by Evenson et al (2011) were developed for hip‐worn accelerometry and counts‐based analysis.

Further, factors associated with overall PA (ie, correlates) and LTPA during pregnancy are not well established. Correlates can be guided by the socioecologic framework, and to date, most correlates explored have focused on the intrapersonal level.^12^ Previous studies have shown that higher education,^13^, ^14^, ^15^ higher income,^8^, ^14^, ^15^ white skin color,^8^, ^13^, ^14^ and PA prior to pregnancy^16^, ^17^, ^18^ have been positively associated with PA and LTPA during pregnancy. Parity^13^, ^14^, ^16^ has been negatively associated with PA during pregnancy. However, divergent associations have been reported for marital status,^14^, ^16^ maternal age,^8^, ^14^ pre‐pregnancy body mass index (BMI),^9^, ^16^, ^18^ history of miscarriage,^13^ preterm birth,^8^ employment during pregnancy,^5^ and smoking status.^13^, ^14^, ^18^

Few studies have assessed PA objectively in combination with a number of potential correlates of PA in large samples of pregnant women. In addition, as far as we know, this is the first study to date to use wrist‐worn accelerometer using raw data in the analysis of physical activity during pregnancy, while all previous studies have used hip‐worn devices and counts‐based analysis. This knowledge is important to inform future interventions aimed at increasing PA among pregnant women. Thus, the purposes of this study were as follows: (a) to describe accelerometer‐assessed overall PA and time spent in moderate PA (MPA), vigorous PA (VPA), and moderate‐to‐vigorous PA (MVPA) during pregnancy; and (b) to identify correlates of PA during pregnancy in women enrolled in the 2015 Pelotas (Brazil) Birth Cohort Study.

## 2 METHODS

### 2.1 Study design

We conducted a population‐based study among pregnant women living in the urban area of Pelotas, a midsized city in southern Brazil with approximately 320 000 inhabitants.^19^ The 2015 Pelotas Birth Cohort Study recruited pregnant women from all health facilities offering antenatal care (public and private) including clinical laboratories, ultrasound clinics, basic health units, hospitals, clinics/polyclinics, colleges, and private doctor offices in the city of Pelotas. The study was designed to include pregnant women with an expected delivery date from 1 January 2015 to 31 December 2015, as part of the antenatal phase of the 2015 Pelotas (Brazil) Birth Cohort Study. Details on the cohort design, recruitment, and enrollment can be found elsewhere.^20^

Three types of questionnaires were administered according to gestational age at enrollment: (a) women identified and enrolled before 16 weeks pregnancy answered the *initial assessment* questionnaire; (b) for women that answered the first questionnaire, a *main assessment* questionnaire was administered between weeks 16 and 24 of gestation; and (c) women who were enrolled after 16 weeks and up to 24 weeks pregnancy responded to a *combined assessment* questionnaire that consisted of a combination of the information collected in the *initial assessment* and *main assessment*. Accelerometry data were collected for those women that answered *main assessment questionnaire* and the *combined assessment questionnaire (groups* 2 *and* 3*)*. For logistical reasons, we collected objectively measured physical activity in the second trimester of pregnancy given that the antenatal follow‐up of the 2015 Pelotas Birth Cohort study also occurred at this time. Questionnaires can be found at http://www.epidemio-ufpel.org.br/site/content/coorte_2015/questionarios.php. Most of the variables in our study were assessed during the antenatal study. However, socioeconomic position (SES), parity, height, pre‐pregnancy weight, and LTPA prior to pregnancy were assessed at the hospital up to 48 h after delivery.

The study was approved by the School of Medicine Ethics Committee of the Federal University of Pelotas in an official letter numbered 522/064. Written informed consent was obtained from all participants.

### 2.2 Physical activity accelerometer‐assessed

Participants were instructed to wear an accelerometer (ActiGraph wGT3X‐BT, Pensacola, Florida, United States) on their non‐dominant wrist in a 24‐h protocol for seven consecutive days. After eight calendar days, accelerometers were collected by the research team at the participant's home or workplace. Accelerometers were programmed to capture data from the first midnight after deployment to midnight seven days later. Participants were instructed not to remove the accelerometer—not even for showering or sleeping. Pregnant women with a walking disability, as well as those working in places where wearing any type of bracelet was not allowed, were excluded from the accelerometry study.

The accelerometer measured the acceleration in three axes (*x, y*, and *z*) within a dynamic range of ± 8 g and a sampling frequency of 30 Hz. Data were stored directly as sampled from the MEMS chip (unfiltered), and raw data were expressed in gravitational equivalent units called milli‐*g* (1000 m*g *= 1 *g* = 9.81 m/s^2^).

### 2.3 Accelerometer data processing and analysis

Accelerometer data were downloaded using ActiLife 6.11.7 software. To identify the best criterion to define the minimum number of adherent days to represent a week, we performed analysis estimating intraclass correlation coefficient of the comparison between different numbers of measurement days (1 to 6 days) and the best scenario of seven complete days of measurement. We used Bland‐Altman plots to evaluate agreement across different PA levels between the different numbers of measurement days and seven complete days. Using the accelerometer for at least four days, the intraclass correlation coefficient was high (>.90) in all categories analyzed compared to seven complete days. Thus, individuals were included in the analyses if data were available for at least four days of total wear time. The following parameters were used to consider adherent data: 24‐h cycle <1, calibration error <0.02, and at least four days of measurement.

Accelerometer raw data analyses were performed in the R package GGIR.^21^, ^22^ Euclidian norm (vector magnitude of the three axes) minus 1 g (ENMO) (calculated as √x^2^+y^2^+z^2^‐1 g) was used to calculate activity‐related acceleration. Activity intensity was estimated as average time per day spent in MVPA from 5‐s aggregated time series. The summary measures used were (a) the average magnitude of wrist acceleration considering the overall volume of body movement, (b) the distribution of time spent across moderate and vigorous intensity levels, and (c) estimated time spent in 5‐ and 10‐min bouts of MVPA. Overall PA was expressed as the daily mean acceleration in m*g* and did not include sedentary behavior, while MVPA was considered as activities with acceleration equal to or higher than 100 m*g*.^23^ MPA was defined as activity with acceleration equal to or higher than 100 m*g* and <400 m*g,* while VPA was defined as activity with acceleration equal or higher than 400 m*g*.^24^ MVPA bouts were defined as consecutive periods in which participants spent at least 80% of time with acceleration equal to or higher than 100 m*g*. Overall PA (non‐bouted) and MVPA in 5‐min bout were the main outcomes in the association analyses. Bouts of MVPA were identified as 5‐ or 10‐min time windows that started with a 5‐s epoch value equal to or higher than 100 m*g* and for which 80% of subsequent 5‐s epoch values were equal to or higher than the 100 m*g* threshold.^23^

### 2.4 Correlates of physical activity

The potential correlates were defined as follows: age (<20;20‐29; 30‐39; ≥40); skin color (white; black/brown/yellow/indigenous), marital status (living with a partner; living without a partner), parity (1; 2; 3; ≥4), schooling (0‐4; 5‐8;9‐11; ≥12 years), paid job during pregnancy (yes/no), height and pre‐pregnancy weight (measured by self‐report), LTPA prior to pregnancy (<150; ≥150 min/week measured by questionnaire), smoking during pregnancy (yes/no—if yes number of cigarettes were assessed),^25^ alcohol use during pregnancy (yes/no), history of miscarriage (yes/no), history of preterm birth (yes/no), and PA advice in prenatal care (yes/no—if yes type of advice was asked). SES was constructed based on a standardized socioeconomic questionnaire^26^ and later categorized into quintiles for analysis. Pre‐pregnancy body mass index (BMI) was calculated by dividing weight by height squared (kg/m^2^), and cutoffs were defined according to the World Health Organization.^27^

### 2.5 Statistical analysis

Descriptive analyses are presented in relative (%) and absolute frequencies. Analysis of variance (anova) and T test or Kruskal‐Wallis and Wilcoxon nonparametric tests were used to compare acceleration mean differences across potential correlates.

Outcome distributions were checked graphically using a histogram and by the parameters mean, median, skewness, and kurtosis. Because of positive skewness, both the 5‐ and 10‐min MVPA variables were log‐transformed for the analyses and presented as a geometric mean. The results for the beta coefficient in crude and adjusted analysis were calculated in exponential function and represented a multiplicative relationship. Unadjusted and adjusted analyses were performed using linear regression.

The potential confounders were evaluated by hierarchical analysis. The hierarchical model consisted of three levels: (a) sociodemographic: age, skin color, marital status, schooling, SES, and paid job during pregnancy; (b) characteristics of reproductive health, anthropometric, and behavioral: parity, pre‐pregnancy BMI, self‐reported LTPA before pregnancy; history of miscarriage or preterm birth, smoking and alcohol use during pregnancy; and (c) PA advice in prenatal care. The backward method was applied for the selection of variables that remained in the regression model. The final model included all variables with *P* < .20. Statistical significance was set at 5%, and 95% confidence intervals were adopted. All analyses were performed using the software Stata version 12.1 (Stata Corporation, College Station, TX, USA).

## 3 RESULTS

A total of 4426 women were interviewed during the antenatal period and 1536 women were interviewed outside of the time window for the accelerometry data collection (between 16 and 24 weeks’ gestation) and therefore excluded from the present analyses. Further, 78 women were additionally excluded (58 did not fulfill the inclusion criteria, 18 miscarried, and 2 stillbirths). In total, 2812 women were eligible to wear the accelerometer. Of these, 31 women refused to participate and 161 were considered lost to follow‐up. Overall, 2620 pregnant women provided accelerometry data. However, 78 did not achieve a complete 24‐h cycle, 61 wore the accelerometer for less than four days, 16 data files were corrupted during analysis, and 2 women wore accelerometers that presented problems in the calibration process. According to our protocol, 2463 participants (87.6% from 2812) had adherent accelerometry data. For the present analyses focused on physical activity correlates, 146 women were excluded because they were assigned to the intervention group in the PAMELA (*Physical Activity for Mothers Enrolled in Longitudinal Analysis)* Study, a randomized controlled trial of exercise nested in the 2015 Birth Cohort.^28^ A total of 2317 individuals’ accelerometry data were analyzed (Figure S1).

The sociodemographic, behavioral, health, and reproductive history characteristics from the total and accelerometry sample are presented in Table S1. The analytical sample with accelerometry presented similar characteristics compared to the total population‐based sample, except for skin color which had more white participants in the accelerometer sample. For the accelerometry sample, a high proportion was between 20 and 29 years of age (49%), white skin color (73%), living with a partner (84%), primiparous (52%), and had 9‐11 complete years of schooling (36%). More than half of the women were not unemployed (51%), did not report LTPA before pregnancy (83%), did not drink alcohol (55%), and did not smoke (91%) during pregnancy. Additionally, almost 50% of the sample was classified as normal pre‐pregnancy BMI, 66% did not receive any PA advice in prenatal care, 82% never had a preterm birth, and 66% never had a miscarriage.

**Figure 1:**
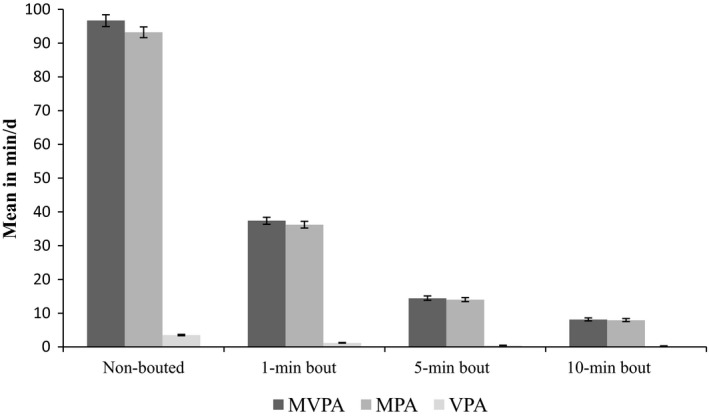
Means of MVPA, MPA, and VPA according to different bout criteria. MVPA, moderate‐to‐vigorous physical activity; MPA, moderate physical activity; VPA, vigorous physical activity

The mean of overall PA was 27.6 m*g* (95% confidence interval (CI): 27.3‐27.9), while the mean of MVPA, 5‐min bout MPA, and VPA was 14 (95% CI: 13.8‐15.1), 14 (95% CI: 13.4‐14.6), and 0.4 (95% CI: 0.4‐0.5) min/day, respectively. The longer bout criteria resulted in lower averages of minutes spent in MVPA, MPA, and VPA (Figure 1).

Table 1 describes the mean of overall PA (mg/s) and 5‐ and 10‐min bouts of MVPA (min/day) stratified by potential correlates. Bivariate analysis showed an inverse association between schooling and SES with means of overall PA. Parous women had higher means of overall PA than nulliparous women. Women that reported receiving advice to not to do physical activity during pregnancy had lower means of overall PA. Similar results were found when analyzing 5‐ and 10‐min bouts of MVPA. In addition, younger women who living without a partner, with other skin color than not white, high schooling and income, normal weight, without use of alcohol during pregnancy, and with history of preterm birth presented higher means of MVPA (5‐ and 10‐min bouts) compared with their pairs.

**Table 1:** Descriptive analysis of physical activity according to potential correlates. The 2015 (Pelotas) Brazil Birth Cohort Study

	Overall PA (m*g*)			MVPA (5‐min bout: min/day)			MVPA (10‐min bout:min/day)		
	Mean	95%CI	*P*	Mean	95%CI	*P*	Mean	95%CI	*P*
Age (years)									
<20	27.8	27.1‐28.6	.07	18.9	17.3‐20.6	**<.001**	11.9	10.6‐13.2	**<.001**
20‐29	27.9	27.4‐28.3		15.2	14.3‐16.2		8.6	7.9‐9.3	
30‐39	27.2	26.6‐27.7		11.6	10.7‐12.6		6.0	5.4‐6.6	
≥40	26.1	24.7‐27.5		12.5	9.1‐15.9		7.6	4.8‐10.4	
Skin color									
White	27.2	26.9‐27.6	**<.001**	13.0	12.3‐13.7	**<.001**	7.1	6.6‐7.6	**<.001**
Black/Brown/Yellow/Indigenous	28.5	27.9‐29.1		18.3	16.9‐19.6		10.9	9.8‐12.0	
Marital status									
Living with a partner	27.5	27.1‐28.9	.27	13.5	12.9‐14.2	**<.001**	7.4	6.9‐7.8	**<.001**
Living without a partner	28.0	27.2‐27.8		19.4	17.5‐21.2		12.3	10.8‐13.8	
Parity									
1 (primaparae)	26.9	26.5‐27.3	**<.001**	14.8	13.7‐15.6	**.001**	8.5	7.8‐9.2	**.001**
2	27.9	27.2‐28.4		12.8	11.7‐14.0		6.8	5.9‐7.6	
3	29.4	28.2‐30.6		16.8	13.9‐19.6		9.2	7.1‐11.4	
≥4	29.2	27.7‐30.7		17.5	14.3‐20.7		10.0	7.8‐12.2	
Schooling (years)									
0‐4	29.9	28.6‐31.1	**<.001**	19.4	16.7‐22.1	**<.001**	11.7	9.7‐13.8	**<.001**
5‐8	28.4	27.8‐29.1		18.2	16.8‐19.7		10.8	9.6‐12.0	
9‐11	27.9	27.4‐28.5		15.2	14.1‐16.2		8.5	7.7‐9.3	
12+	26.0	25.6‐26.6		10.2	9.4‐10.9		5.3	4.7‐5.8	
SES (quintiles)									
Q1(poorest)	29.6	28.6‐30.5		21.4	19.0‐23.8	**<.001**	13.1	11.3‐15.0	**<.001**
Q2	29.1	28.3‐29.8		18.9	17.2‐20.7		10.9	9.4‐12.2	
Q3	27.8	27.1‐28.5		13.8	12.5‐15.1		7.5	6.5‐8.4	
Q4	26.7	26.0‐27.3		11.8	10.7‐13.0		6.2	5.4‐7.1	
Q5 (wealthiest)	25.6	25.0‐26.2		8.8	7.7‐9.9		4.6	3.8‐5.4	
Paid job during pregnancy									
Yes	28.3	27.9‐28.8	**<.001**	13.4	12.6‐14.2	**.001**	7.2	6.6‐7.9	**<.001**
No	26.8	26.4‐27.2		15.5	14.6‐16.4		9.0	8.3‐9.7	
Self‐reported pre‐pregnancy LTPA (minutes/week)									
≥150	27.6	26.8‐28.4	.98	15.6	13.9‐17.4	.10	9.2	7.8‐10.6	.06
<150	27.6	27.2‐28.0		14.2	13.5‐15.0		7.9	7.3‐8.4	
Pre‐pregnancy body mass index (kg/m^2^)									
Underweight (<18.5)	27.9	26.1‐29.6	.51	15.7	11.9‐19.4	**.02**	8.4	5.8‐11.0	**.01**
Normal (18.5‐24.9)	27.8	27.3‐28.3		15.2	14.2‐16.1		8.7	7.9‐9.4	
Overweight (25‐29.9)	27.6	27.0‐28.2		13.4	12.3‐14.6		7.3	6.4‐8.2	
Obese (≥30)	27.1	26.5‐27.8		12.7	11.4‐14.1		7.0	6.0‐7.9	
Smoking during pregnancy (number of cigarettes/day)									
Non‐smoker	27.2	26.8‐27.6	.84	13.4	12.6‐14.2	.66	7.3	6.7‐7.9	.74
<5	28.0	25.5‐30.5		16.5	9.8‐23.4		8.9	3.6‐14.1	
5‐9	28.4	26.4‐30.4		13.2	9.5‐16.8		6.7	3.9‐9.4	
10‐15	26.6	24.3‐28.9		12.4	8.4‐16.5		6.3	3.3‐9.2	
>15	27.7	24.5‐30.8		14.7	8.9‐20.4		9.0	4.4‐13.6	
Alcohol use during pregnancy									
Yes	27.7	27.3‐28.2	.37	14.1	13.3‐15.0	**.04**	8.6	7.9‐9.3	**.03**
No	27.5	27.0‐27.9		14.9	14.0‐15.8		7.8	7.2‐8.4	
History of miscarriage									
Yes	27.7	27.0‐28.4	.31	13.9	12.9‐15.1	.96	7.6	6.5‐8.6	.94
No	28.2	27.7‐28.7		14.0	12.5‐15.3		7.7	6.9‐8.5	
History of preterm birth									
Yes	28.9	27.9‐29.8	.05	15.6	13.5‐17.7	**.02**	8.8	7.3‐10.4	**.01**
No	27.8	27.3‐28.2		13.5	12.6‐14.5		7.3	6.7‐8.0	
PA advice in prenatal care									
No	28.1	27.7‐28.5	**<.001**	15.6	14.7‐16.4	**<.001**	8.9	8.2‐9.5	**<.001**
Yes—should to do PA	26.8	26.2‐27.3		13.0	12.0‐14.1		7.3	6.4‐8.1	
Yes—should to change or to decrease PA	26.3	24.5‐28.1		10.1	7.5‐12.7		5.3	3.5‐7.2	
Yes—should not to do PA	23.6	22.1‐25.1		6.7	4.6‐8.8		3.0	1.5‐4.6	

MVPA, moderate‐to‐vigorous intensity physical activity; LTPA, leisure‐time physical activity; PA, physical activity; min/day, minutes/day; CI, confidence interval; m*g,* gravitational equivalent expressed in milli‐*g* (1000 m*g *= 1 *g *= 9.81 m/s^2^). anova for ordinal variables and t test for categorical variables. Nonparametric Kruskal‐Wallis test for ordinal variables and Wilcoxon test for dichotomized variables.

For the adjusted analysis, we performed the linear regression using both MVPA bouts criteria (5‐ and 10‐min bouts). The results were very similar, so we chose 5‐min bout to present because the log transformation was more normally distributed than the 10‐min bout (Table 2). An inverse linear association between MVPA and age was observed. Women 40 years and older had lower means of MVPA (β: 0.70; CI 95%: 0.51; 0.97) compared to women <20 years. MVPA means were 21% higher in women with other skin color compared to white women. Time spent in MVPA was significantly lower in women living with a partner (β: 0.83; 95% CI: 0.72; 0.94) compared to women living without a partner.

**Table 2:** Crude and adjusted association between overall PA and MVPA in 5‐min bout according to potential correlates. The 2015 Pelotas (Brazil) Birth Cohort Study

	Overall PA (m*g*)						MVPA (5‐min bout: min/day)					
	Crude			Adjusted^c^			Crude			Adjusted^d^		
	β	95%CI	*P*	β	95%CI	*P*	β^e^	95%CI	*P*	β^e^	95%CI	*P*
Age (years)												
<20	**‐**	**‐**	.07^a^	**‐**	**‐**	.92^a^	‐	‐	**<.001** a	‐	‐	**.001** a
20‐29	0.03	−0.89; 0.94		0.63	−0.42; 1.67		0.74	0.65; 0.84		0.84	0.72; 0.99	
30‐39	−0.67	−1.63; 0.29		0.66	−0.47; 1.79		0.57	0.49; 0.65		0.76	0.63; 0.90	
≥40	−1.75	−3.76; 0.26		−1.25	−3.40; 0.90		0.61	0.45; 0.81		0.70	0.51; 0.97	
Skin color												
White	**‐**	**‐**	**<.001**	**‐**	**‐**	.45	‐	‐	**<.001**	‐		**.001**
Black/Brown/yellow/indigenous	1.23	0.55; 1.91		0.29	−0.46; 1.04		1.45	1.31; 1.58		1.21	1.08; 1.36	
Marital status												
Living with a partner	−0.47	−1.30; 0.37	.27	0.48	−0.42; 1.38	.30	0.68	0.60; 0.77	**<.001**	0.83	0.72; 0.94	**.006**
Living without a partner	‐	**‐**		‐	‐		‐	‐		‐	‐	
Parity												
1 (primaparae)			**<.001** b			.09^b^			**.008** b			.40^b^
2	0.93	0.19; 1.67		0.72	−0.19; 1.64		0.85	0.76; 0.95		0.99	0.75; 1.36	
3	2.50	1.36; 3.63		1.21	−0.31; 2.73		1.04	0.87; 1.23		1.08	0.76; 1.55	
≥4	2.31	0.90; 3.73		0.77	−1.08; 2.62		1.14	0.92; 1.40		1.14	0.76; 1.68	
Schooling (years)												
0‐4	**‐**	**‐**	**<.001** a	**‐**	**‐**	**<.001** a	‐	‐	**<.001** a	‐	‐	**.001** a
5‐8	−1.44	−2.68; −0.21		−1.81	−3.18; −0.43		0.95	0.79; 1.13		0.95	0.78; 1.17	
9‐11	−1.95	−3.11; −0.79		−2.86	−4.18 −1.53		0.81	0.68; 0.95		0.86	0.71; 1.05	
12+	−3.78	−4.95; −2.62		−4.35	−5.82; −2.89		0.54	0.45; 0.64		0.73	0.58; 0.90	
SES (quintiles)												
Q1(poorest)	‐	**‐**	**<.001** a	**‐**	**‐**	**<.001** a	‐	‐	**<.001** a	‐	‐	**<.001** a
Q2	−0.49	−1.60; 0.63		−0.31	−1.41; 0.79		0.91	0.78; 1.08		1.00	0.85; 1.17	
Q3	−1.76	−2.86; −0.66		−1.51	−2.64; −0.39		0.67	0.57; 0.79		0.76	0.65; 0.90	
Q4	−2.91	−4.00; −1.81		−2.51	−3.68; −1.35		0.59	0.50; 0.69		0.72	0.60; 0.85	
Q5 (wealthiest)	−3.97	−5.07; −2.87		−3.47	−4.72; −2.22		0.42	0.36; 0.50		0.56	0.46; 0.68	
Paid job during pregnancy												
Yes	1.51	0.91; 2.12	**<.001**	3.13	2.41; 3.84	**<.001**	0.90	0.82; 0.98	**.01**	1.16	1.05;1.30	**.005**
No	**‐**	**‐**		‐	‐		**‐**	**‐**		‐	‐	
Self‐reported pre‐pregnancy LTPA (minutes/week)												
≥150	−0.009	−0.89; 0.87	.98	0.06	−1.00; 1.12	.92	1.12	0.98; 1.28	.08	1.06	0.84; 1.35	.63
<150	**‐**	**‐**		**‐**	**‐**		‐	‐		‐	‐	
Pre‐pregnancy body mass index (kg/m^2^)												
Underweight (<18.5)	**‐**	**‐**	.51^b^	**‐**	**‐**	.07^b^	‐	‐	.05^b^	‐	‐	.39^b^
Normal (18.5‐24.9)	−0.08	−1.89; 1.72		0.80	−1.70; 3.30		0.96	0.74; 1.25		1.23	0.68; 2.23	
Overweight (25‐29.9)	−0.25	−2.10; 1.59		0.002	−2.55; 2.55		0.84	0.64; 1.11		1.11	0.61; 1.99	
Obese (≥30)	−0.75	−2.64; 1.15		−0.32	−2.93; 2.29		0.83	0.63; 1.07		1.09	0.60; 1.99	
Smoking during pregnancy (number of cigarettes/day)												
Non‐smoker	**‐**	**‐**	.84^b^	**‐**	**‐**	.29^b^	‐	‐	.74^b^	‐	‐	.07^b^
<5	0.78	−1.58; 3.14		−0.21	−2.76; 2.33		1.22	0.87; 1.70		1.01	0.65; 1.58	
5‐9	1.17	−1.58; 3.93		0.49	−2.41; 3.39		1.16	0.77; 1.75		0.88	0.50; 1.55	
10‐15	−0.66	−3.33; 2.00		−1.42	−4.28; 1.44		0.99	0.66; 1.46		0.77	0.49; 1.22	
>15	0.43	−2.80; 3.66		−1.53	−4.84; 1.77		1.12	0.70; 1.77		0.63	0.35; 1.12	
Alcohol use during pregnancy												
Yes	0.28	−0.33; 0.89	.37	0.34	−0.49; 1.17	.43	1.09	1.00; 1.20	.05	0.97	−0.21; 1.16	.73
No	**‐**	**‐**		**‐**	**‐**		‐	‐		‐	‐	
History of miscarriage												
Yes	−0.44	−1.30; 0.42	.31	0.55	−0.63; 1.74	0.36	1.04	0.91; 1.19	.53	1.16	−0.03; 1.38	.11
No	**‐**	**‐**		**‐**	**‐**		‐	‐		**‐**	**‐**	
History of preterm birth												
Yes	1.08	0.02; 2.13	.05	1.11	−0.39; 2.62	.15	1.11	0.95; 1.30	.19	1.13	−0.10; 1.42	.29
No	**‐**	**‐**		**‐**	**‐**		‐	‐		**‐**	**‐**	
PA advice in prenatal care												
No	**‐**	**‐**	**<.001** b	**‐**	**‐**	**.02** b	‐	‐	**<.001** a	‐	‐	.06^a^
Yes—should to do PA	−1.39	−2.07; −0.72		−0.27	−0.17; 0.63		0.87	0.79; 0.96		0.91	0.75; 1.12	
Yes—should to change or to reduce PA	−1.82	−3.98; 0.33		−0.06	−2.52; 2.39		0.77	0.79; 1.07		1.11	0.60; 2.01	
Yes—should not to do PA	−4.52	−6.42; −2.62		−3.59	−5.86; −1.31		0.52	0.56; 0.70		0.57	0.34; 0.93	

MVPA, moderate‐to‐vigorous intensity physical activity; LTPA, leisure‐time physical activity; SES, socioeconomic status; PA, physical activity; min/day = minutes/day; CI, confidence interval m*g*; gravitational equivalent expressed in milli‐*g* (1000 m*g *= 1 *g* = 9.81 m/s^2^).

a Test of linear trend.

b Test of heterogeneity.

c Adjusted for SES, schooling, paid job during pregnancy, parity smoking during pregnancy and PA advice during prenatal care.

d Adjusted for age, skin color, marital status, SES, schooling, paid job during pregnancy, smoking during pregnancy, history of miscarriage and preterm birth and PA advice during prenatal care.

e Analyses were performed using log‐transformed, and the results are presented as geometric means.

Schooling and income were inversely associated with MVPA (Table 2). Women with the highest education and income categories spent 27% and 44% less min/day in MVPA, respectively, compared to women in the lowest schooling and income categories. Working women presented higher means of MVPA compared to those unemployed. Parity and self‐reported pre‐pregnancy BMI were not associated with MVPA after adjustment for confounders. Women that reported receiving advice to not to do exercising during pregnancy spent 43% less time in MVPA compared to women that reported not receiving any counseling to do physical activity.

Years of education and income were inversely associated with overall PA (Table 2). Women who worked during pregnancy recorded higher overall PA, while women that reported receiving advice to not exercise during pregnancy presented lower overall PA (β: −3.59; CI 95%: −5.86; −1.31) compared to women that reported not receiving any counseling to do physical activity.

## 4 DISCUSSION

We assessed objectively measured PA and examined associations with potential correlates of PA during pregnancy in more than 2000 pregnant women belonging to a population‐based cohort study in southern Brazil. Our findings showed low levels of PA; women spent on average 14 min/day in MVPA and 0.4 min/day in VPA, which was similar to the national U.S. estimates from 2003 to 2006.^8^ Differences were found across sociodemographic groups and by type of PA advice in prenatal care. Means of MVPA between extremes of age, schooling, and income were 25, 45, and 84 min/week, respectively. The magnitude of these differences is clinically relevant because it may influence these specific groups to meet the current recommendations of weekly physical activity.

In general, we observed low levels of PA during pregnancy, especially considering vigorous activities. This study found that larger bout criterion (ie, 10 min vs 5 min) resulted in smaller averages of MVPA, MPA, and VPA. The discussion on bouts is important because bout criteria might change the PA constructs which have been assessed. The longer the bout, the less likely individuals will accumulate time in PA. However, this methodological decision might be important to collect data regarding a more structured exercise or PA behavior. Otherwise, intermittent exercise, especially for VPA estimates, is an example that could not be captured with a larger bout window because the majority of these activities are performed in less than five minutes at a time or not performed at all. Defining the exact duration of a bout remains a challenge when measuring PA by accelerometry. In the present analyses, associations between correlates of PA and PA were not affected by different definitions of the bout length.

Contrary to previous results,^8^ we observed an inverse association between age and MVPA, but the same association was not confirmed in a study using hip accelerometry and analyzed in counts/min.^8^ Previous studies in pregnant women using self‐reported PA measures have reported conflicting results for the association between PA and age.^13^, ^14^ Women of non‐white skin color and women living without a partner spent consistently more time in MVPA. These observations differ from those previously published^8^, ^14^ and may be explained by social inequalities. Social determinants shape the health profile of the population and the adoption of health‐related behaviors. In Brazil, black skin color and marital status are associated with lower income and education. In our study, some residual confounding may persist even after adjusted analysis. Another possible hypothesis is that women living with a partner can be preserved from daily activities during pregnancy being the most part of the heavy physical activities perform by the partner.

The socioeconomic covariates previously reported that are positively correlated with total PA, MVPA, and LTPA included employment during pregnancy,^5^ higher education,^13^, ^14^ and higher income.^8^, ^14^ In contrast, when analyzed by accelerometry, we observed an inverse association between PA (both overall PA and MVPA) and socioeconomic position and years of education. These differences can be explained because accelerometer‐assessed physical activity considers a global measure of PA including four domains (household, occupational, leisure, and commuting), while self‐reported PA usually focused solely on LTPA. In low‐ and middle‐income countries such as Brazil, poorer people are more physically active at work, in household activities, and commuting,^29^ which can explain why objective measures of PA are associated with the lowest income and education quintiles. Nonetheless, few studies have used these methods to measure PA during pregnancy, especially in low‐ and middle‐income countries.

The majority of the available evidence of correlates related to PA during pregnancy are based on self‐reported measures focused solely on LTPA and mostly are derived from high‐income settings. Therefore, caution should be taken in the comparison of our findings with previous studies because of cultural and socioeconomic differences, as well as the different constructs of physical activity that are ascertained and performed. These discrepancies highlight the contribution objective methods can make when examining correlates of PA, especially in low‐ and middle‐income countries such as Brazil, where a significant proportion of total PA takes place in commuting, household, and occupational domain compared to LTPA.^23^ Moreover, it is important to emphasize that this difference between high‐ vs low/middle‐income countries regarding the socioeconomic factors and PA association is also observed in the general population and not only in pregnant women.^23^

Prior studies^16^, ^17^, ^18^ have shown that LTPA before pregnancy may be a strong predictor of PA during pregnancy. While studies found that LTPA prior to pregnancy was positively associated with exercise during pregnancy,^16^, ^17^, ^18^ findings for pre‐pregnancy BMI,^16^, ^18^ alcohol use, and smoking^13^, ^14^, ^16^, ^18^ have been less consistent. In contrast to other studies,^9^, ^16^, ^18^ we observed that pre‐pregnancy BMI was not associated with overall PA and MVPA. Similar to previous studies,^13^, ^14^ no clear PA patterns were observed for smoking and alcohol use during pregnancy. Also, we did not observe any association between LTPA before pregnancy and overall PA. Time spent in MVPA was similar among those women who were active and not active before pregnancy. The possible hyphotesis for these discrepancies with other studies it is because self‐report LTPA (positively) as well other behavioral aspects such as alcohol use and smoking (negatively) are strongly associated with socioeconomic levels in middle‐income countries such as Brazil.^15^, ^30^, ^31^ In our analysis, the potential correlates were evaluated by hierarchical levels based on a theoretical model of causal effects on the associations related to PA during pregnancy and to attempt to eliminate problems related to confounding factors.

Similar to one previous study,^8^ we did not observe any differences in PA between those with a history of preterm birth or miscarriage. Given the current evidence of the potential benefits of PA during pregnancy on maternal and child health,^1^ especially on the prevention of preterm birth,^1^, ^32^ it is possible that women with a history of preterm birth are including more active behaviors in their daily routine. It is important to acknowledge that history of miscarriage and preterm birth were based on self‐report, and it might lead to misclassification.

Our findings were inconsistent with previous studies that observed an inverse association between parity and PA during pregnancy.^5^, ^13^, ^14^, ^16^ A possible explanation for this disagreement is because most these previous studies focused on LTPA. PA by accelerometry considers all domains of PA. Mothers with more than one child probably have to be more physically active during childcare and spend more time involved with children's routine including transportation and household activities. However, this association between overall PA and MVPA and number of children was attenuated in the adjusted analysis.

Pregnant women that reported receiving advice from health providers to stop exercising during pregnancy presented lower overall PA and MVPA compared to other categories. This finding highlights the importance of provider advice during prenatal care. Among women without complications, health providers should focus on encouraging continued PA during pregnancy among those who are already active and should specifically target PA promotion among those women performing irregular or no activity.^14^

To the best of our knowledge, this is the first study to date to use wrist‐worn accelerometer using raw data in the analysis of physical activity during pregnancy. The most significant strengths of our study are the high response rate, the large and diverse population‐based sample, the detailed information on a number of potential correlates, and the use of accelerometry for assessing PA. Analysis from raw accelerometry data allows for transparency in all stages of data processing and enhanced in the near future a higher comparability between data collected from different accelerometer brands. Furthermore, wrist‐worn accelerometry provides improvements on participant's compliance compared to accelerometers worn at the hip or ankle,^33^ and previous studies have used the same methodological approach and have reported the advantages to using this parameterization.^23^, ^24^

Some limitations should be considered as well. Swimming, cycling, and any weight‐bearing PA (ie, lifting weights) are not adequately captured by accelerometers and may underestimate PA levels. Accelerometers alone cannot easily identify the domain in which PA was performed, which makes it impossible to discriminate the four domains of PA: household, occupational, commuting, and leisure activities.^34^, ^35^ Specifically regarding the accelerometer placement, the lower number of studies using wrist‐worn accelerometry limits comparability of our findings. Also, wrist placement may overestimate PA due to upper limb movement without corresponding increases in energy expenditure. There are few studies directly comparing two placement sites using the GT3X, and they have shown that wrist placement may produce less accurate classification of PA intensity compared to the hip in free‐living conditions.^36^, ^37^ However, there is also other evidence showing similar validity parameters between wrist accelerometry and hip accelerometry.^33^

In summary, the current study indicated low levels of PA measured by accelerometry in a population‐based sample of pregnant women from Brazil. Correlates of overall and MVPA included sociodemographic factors and PA advice in prenatal care.

### 4.1 Perspectives

Although the effectiveness of PA on improving maternal and neonatal outcomes has been examined, the correlates and patterns of PA during pregnancy have not been assessed using objective measures. The identified correlates may provide a framework to better understand factors that influence PA in pregnant women and thus inform future interventions aimed at increasing PA levels during pregnancy. These findings may be useful to establish population subgroups in need for intervention, and it may provide evidence for future recommendations for PA during pregnancy.

## COMPETING INTERESTS

The authors declare that they have no competing interests.
