# Supplementary material for: Correlates of accelerometer‐assessed physical activity in pregnancy—The 2015 Pelotas (Brazil) Birth Cohort Study
Source: Scand J Med Sci Sports. 2018 Apr 6;28(8):1934–45. doi: 10.1111/sms.13083 (PMC6055654; doi:10.1111/sms.13083)
Supplement: Supplementary file 1 [file SMS-28-1934-s001.docx]

| **Table S1.** Original and analytical sample according to sociodemographic, behavior, health and reproductive history. The 2015 (Pelotas) Brazil Birth Cohort Study. | | | | | | |
| --- | --- | --- | --- | --- | --- | --- |
|  | **Antenatal study**  **(n=4,426)** | |  | **Accelerometry sample (n=2,317)** | |  |
|  | **N** | **%** |  | **n** | **%** | ***p*** |
| **Age (years)** |  |  |  |  |  | 0.95 |
| <20 | 638 | 14.4 |  | 325 | 14.0 |  |
| 20-29 | 2,164 | 48.9 |  | 1,135 | 49.0 |  |
| 30-39 | 1,510 | 34.1 |  | 793 | 34.2 |  |
| ≥ 40 | 114 | 2.6 |  | 64 | 2.8 |  |
| **Skin color** |  |  |  |  |  | **0.04** |
| White | 3,114 | 70.4 |  | 1,686 | 72.9 |  |
| Black/Brown/Yellow/Indigenous | 1,307 | 29.6 |  | 628 | 27.1 |  |
| **Marital Status** |  |  |  |  |  | 0.30 |
| Living with a partner | 3,690 | 83.4 |  | 1,955 | 84.4 |  |
| Living without a partner | 736 | 16.6 |  | 362 | 15.6 |  |
| **Parity** |  |  |  |  |  | 0.72 |
| 1 (primaparae) | 1,614 | 51.9 |  | 968 | 51.8 |  |
| 2 | 994 | 31.2 |  | 600 | 32.1 |  |
| 3 | 337 | 10.6 |  | 187 | 10.1 |  |
| ≥ 4 | 209 | 6.3 |  | 113 | 6.1 |  |
| **Schooling (years)** |  |  |  |  |  | 0.53 |
| 0-4 | 370 | 8.4 |  | 190 | 8.2 |  |
| 5-8 | 995 | 22.5 |  | 487 | 21.0 |  |
| 9-11 | 1563 | 35.3 |  | 844 | 36.4 |  |
| 12+ | 1496 | 33.8 |  | 796 | 34.4 |  |
| **SES (quintiles)** |  |  |  |  |  | 0.69 |
| Q1(poorest) | 511 | 16.7 |  | 276 | 15.2 |  |
| Q2 | 618 | 20.2 |  | 367 | 20.2 |  |
| Q3 | 644 | 21.1 |  | 386 | 21.2 |  |
| Q4 | 645 | 21.0 |  | 397 | 21.8 |  |
| Q5 (wealthiest) | 641 | 21.0 |  | 394 | 21.7 |  |
| **Paid job during pregnancy** |  |  |  |  |  | 0.24 |
| Yes | 1,954 | 47.3 |  | 1,130 | 48.8 |  |
| No | 2,180 | 52.7 |  | 1,185 | 51.2 |  |
| **Self-reported pre-pregnancy LTPA (minutes/week)** |  |  |  |  |  | 0.92 |
| ≥150 | 532 | 16.9 |  | 318 | 17.0 |  |
| <150 | 2,621 | 83.1 |  | 1,550 | 83.0 |  |
| **Pre-pregnancy body mass index (kg/m^2^)** |  |  |  |  |  | 0.78 |
| Underweight (<18.5) | 124 | 3.1 |  | 70 | 3.4 |  |
| Normal (18.5-24.9) | 1,939 | 48.9 |  | 997 | 48.2 |  |
| Overweight (25-29.9) | 1,116 | 28.2 |  | 603 | 29.2 |  |
| Obese (≥30) | 784 | 19.8 |  | 398 | 19.2 |  |
| **Smoking during pregnancy**  **(number of cigarettes/day)** |  |  |  |  |  | 0.98 |
| Nonsmoker | 1,951 | 92.0 |  | 1,290 | 91.4 |  |
| <5 | 57 | 2.7 |  | 40 | 2.8 |  |
| 5-9 | 41 | 1.9 |  | 29 | 2.1 |  |
| 10-15 | 42 | 2.0 |  | 31 | 2.2 |  |
| >15 | 29 | 1,4 |  | 21 | 1.5 |  |

|  | **Antenatal sample**  **(n=4,426)** | |  | **Accelerometry sample (n=2,317)** | |  |
| --- | --- | --- | --- | --- | --- | --- |
|  | **N** | **%** |  | **n** | **%** | ***p*** |
| **Alcohol use during pregnancy** |  |  |  |  |  | 0.92 |
| Yes | 1,841 | 44.5 |  | 1,035 | 44.7 |  |
| No | 2,292 | 55.5 |  | 1,279 | 55.3 |  |
| **History of miscarriage** |  |  |  |  |  | 0.21 |
| Yes | 778 | 32.2 |  | 433 | 34.3 |  |
| No | 1,636 | 67.8 |  | 828 | 65.7 |  |
| **History of preterm birth** |  |  |  |  |  | 0.27 |
| Yes | 404 | 16.8 |  | 230 | 18.2 |  |
| No | 2,007 | 83.2 |  | 1,030 | 81.8 |  |
| **PA advice in prenatal care** |  |  |  |  |  | 0.78 |
| No | 2,672 | 65.2 |  | 1,523 | 66.3 |  |
| Yes – should do PA | 1,222 | 29.8 |  | 667 | 29.0 |  |
| Yes – should to change or to decrease PA | 95 | 2.3 |  | 47 | 2.1 |  |
| Yes - should not to do PA | 109 | 2.7 |  | 61 | 2.7 |  |
| *LTPA = leisure time physical activity; SES: socioeconomic status..  *SES, parity and self-reported LTPA before pregnancy presented a different “n” because 448 women with accelerometry data did not attend the perinatal follow-up.  *Variable with higher number of missing in antenatal sample: pre-pregnancy body mass index (BMI) (n=463)  *Variable with higher number of missing in accelerometry sample: pre-pregnancy BMI (n=249) | | | | | | |

Women interviewed during antenatal study

**n=4,426**

Excluded women

58 = exclusion criteria

18 = miscarried

2 = still births

**1536** women were interviewed out of time window for accelerometry data collection of (16^th^ to 24^th^ weeks of gestation)

Eligible women to accelerometry

**n=2,812**

161 = lost to follow-up

31 = refusals

Accelerometer data

**n=2,620**

78 = complete 24h cycle <1

61 = wear time < 4 days

13 = corrupted files

3 = clipping score issues

2 = calibration error >0.02

Accelerometer valid data

**n=2,463**

146 = excluded of analyses (women belonging to the intervention group in the PAMELA trial

**Final sample**

**n=2,317**

**Figure S1.** Flow-chart describing accelerometry data collection during antenatal study.
